# Predictors of switch to and early outcomes on third-line antiretroviral therapy at a large public-sector clinic in Johannesburg, South Africa

**DOI:** 10.1186/s12981-018-0196-9

**Published:** 2018-04-10

**Authors:** Denise Evans, Kamban Hirasen, Rebecca Berhanu, Given Malete, Prudence Ive, David Spencer, Sharlaa Badal-Faesen, Ian M. Sanne, Matthew P. Fox

**Affiliations:** 1 Health Economics and Epidemiology Research Office, Department of Internal Medicine, School of Clinical Medicine, Faculty of Health Sciences, University of the Witwatersrand, Johannesburg, South Africa; 2 Division of Infectious Diseases, University of North Carolina, Chapel Hill, NC USA; 3 Clinical HIV Research Unit, Department of Internal Medicine, School of Clinical Medicine, Faculty of Health Sciences, University of the Witwatersrand, Johannesburg, South Africa; 4 Right to Care, Johannesburg, South Africa; 5 Department of Global Health, Boston University School of Public Health, Boston, MA USA; 6 Department of Epidemiology, School of Public Health, Boston University, Boston, MA USA

**Keywords:** Third-line ART, Virologic response, Resource-limited setting, South Africa

## Abstract

**Background:**

While efficacy data exist, there are limited data on the outcomes of patients on third-line antiretroviral therapy (ART) in sub-Saharan Africa in actual practice. Being able to identify predictors of switch to third-line ART will be essential for planning for future need. We identify predictors of switch to third-line ART among patients with significant viraemia on a protease inhibitor (PI)-based second-line ART regimen. Additionally, we describe characteristics of all patients on third-line at a large public sector HIV clinic and present their early outcomes.

**Methods:**

Retrospective analysis of adults (≥ 18 years) on a PI-based second-line ART regimen at Themba Lethu Clinic, Johannesburg, South Africa as of 01 August 2012, when third-line treatment became available in South Africa, with significant viraemia on second-line ART (defined as at least one viral load ≥ 1000 copies/mL on second-line ART after 01 August 2012) to identify predictors of switch to third-line (determined by genotype resistance testing). Third-line ART was defined as a regimen containing etravirine, raltegravir or ritonavir boosted darunavir, between August 2012 and January 2016. To assess predictors of switch to third-line ART we used Cox proportional hazards regression among those with significant viraemia on second-line ART after 01 August 2012. Then among all patients on third-line ART we describe viral load suppression, defined as a viral load < 400 copies/mL, after starting third-line ART.

**Results:**

Among 719 patients in care and on second-line ART as of August 2012 (with at least one viral load ≥ 1000 copies/mL after 01 August 2012), 36 (5.0% over a median time of 54 months) switched to third-line. Time on second-line therapy (≥ 96 vs. < 96 weeks) (adjusted Hazard Ratio (aHR): 2.53 95% CI 1.03–6.22) and never reaching virologic suppression while on second-line ART (aHR: 3.37 95% CI 1.47–7.73) were identified as predictors of switch. In a separate cohort of patients on third-line ART, 78.3% (47/60) and 83.3% (35/42) of those in care and with a viral load suppressed their viral load at 6 and 12 months, respectively.

**Conclusions:**

Our results show that the need for third-line is low (5%), but that patients’ who switch to third-line ART have good early treatment outcomes and are able to suppress their viral load. Adherence counselling and resistance testing should be prioritized for patients that are at risk of failure, in particular those who never suppress on second-line and those who have been on PI-based regimen for extended periods.

**Electronic supplementary material:**

The online version of this article (10.1186/s12981-018-0196-9) contains supplementary material, which is available to authorized users.

## Background

In many resource-limited settings (RLS), antiretroviral therapy (ART) treatment guidelines call for switching patients who fail non-nucleoside reverse transcriptase inhibitor (NNRTI)-based first-line regimens to protease-inhibitor (PI) based second-line regimens [1, 2]. Estimates from sub-Saharan Africa in particular suggest that 6% of all patients receiving first-line ART will require second-line treatment per year [3]. However, when looking specifically at South Africa, this is estimated to be closer to 10% [4].

While the availability of second-line ART is expanding in RLS, only a few countries in these settings have treatment options for patients who fail both NNRTI and PI-based first and second-line therapies respectively [4]. Yet as treatment programs mature, an increasing number of patients are now failing second-line therapy [5, 6] forcing programs to make difficult decisions around whether or not to provide expensive third-line regimens. While the absolute number of patients failing second-line may be low, rates of second-line failure have been demonstrated to be as high as 38% by 36 months [7], a number that is likely an underestimate as limited viral load monitoring and genotyping has potentially led to under-reporting of treatment failure [8].

Since 2009 the World Health Organization (WHO) has recommended that third-line regimens be made available in all countries [9], however few are able to provide these regimens due to the high cost and complexity of implementation [6]. Third-line regimens include drugs such as newer generation NNRTIs like etravirine (ETV), boosted PIs like ritonavir boosted darunavir (DRV/r), as well as the integrase inhibitor raltegravir (RAL) [10]. Third-line ART has been estimated to be fifteen times more expensive than first-line and six times more expensive than second-line ART at current prices [11]. Current approaches to third-line therapy also require access to routine viral load monitoring and HIV resistance testing. Unlike the implementation of standardized first- and second-line regimens currently in use in most countries, third-line regimens are generally individualized to account for treatment history, toxicities as well as resistance patterns and therefore require a higher cadre of health care workers.

South Africa is one of the few countries in sub-Saharan Africa that has access to both resistance testing for patients failing second-line, as well as third-line drugs such as ETV, DRV/r and RAL in the public sector. As such we sought to use data from one of the largest cohorts of patients on second-line treatment in sub-Saharan Africa that also had access to third-line. This enabled us to identify predictors of switching to third-line ART among those with significant viraemia on second-line therapy as well as describe early outcomes of patients on third-line ART.

## Methods

### Study site

We conducted a retrospective cohort study among adults (≥ 18 years) attending Themba Lethu Clinic in Johannesburg, South Africa. The Themba Lethu Clinic cohort, which has been described in detail elsewhere [12, 13], is an urban public-sector HIV treatment site, and follows the South African National Department of Health (NDoH) adult ART treatment guidelines [14–17] (summarized in Table 1). Since the start of the national program in April 2004, more than 50,000 adults have been part of the Themba Lethu cohort, of whom close to 40,000 have started on first-line and over 3000 on second-line ART. In August 2012, Themba Lethu Clinic received access to third-line drugs and resistance testing through donor funding. Later, in 2013, access to resistance testing and third-line ART became available in the public sector.

**Table 1:** Summary and comparison of the South African adult ART guidelines

	2004	2010	2012/2013	2015
Eligible to start	CD4 ≤ 200 cells/mm^3^ WHO stage IV	CD4 count ≤ 200 cells/mm^3^ WHO stage IV, MDR or XDR TB if CD4 ≤ 350 cells/mm^3^ Pregnant women with CD4 ≤ 350 cells/mm^3^ or WHO stage III/IV	CD4 count ≤ 350 cells/mm^3^ WHO stage III or IV TB Pregnant or breastfeeding, irrespective of CD4	CD4 count ≤ 500 cells/mm^3^ Active TB disease, HIV positive and pregnant or breastfeeding, known HBV co-infection irrespective of CD4 count or clinical stage WHO stage III or IV irrespective of CD4 count
First-line ART regimen	d4t + 3TC + EFV d4t + 3TC + NVP	TDF + 3TC/FTC + EFV/NVP d4t + 3TC + EFV/NPV AZT + 3TC + EFV/NVP	TDF + 3TC/FTC + EFV/NVP AZT + 3TC + EFV/NVP d4t + 3TC + EFV/NVP ABC + 3TC + EFV/NVP	TDF + 3TC/FTC + EFV TDF + 3TC/FTC + NVP/LPV/r ABC + 3TC + EFV/NVP
Failing first-line	No resistance testing for first-line failure			
Second-line ART regimen	AZT/ddI/LPV/r	TDF + 3TC/FTC + LPV/r AZT + 3TC + LPV/r	AZT + 3TC + LPV/r TDF + 3TC/FTC + LPV/r Switch LPV/r to ATV/r with dyslipidaemia	AZT + 3TC + LPV/r AZT + TDF + 3TC + LPV/r [if Hep B co-infected) TDF + 3TC/FTC + LPV/r Switch LPV/r to ATV/r with dyslipidaemia
Failing second-line/third-line ART		Specialist referral	Specialist referral for regimen with raltegravir/darunavir/etravirine	Documented PI resistance Refer to expert committee for possible darunavir-ritonavir, raltegravir, etravirine or some combination
Monitoring	CD4 and VL at baseline and 6 monthly ALT for patients on NVP at baseline, 2, 4 and 8 weeks and 6 monthly thereafter FBC at baseline, monthly for 3 month and then 6 monthly Fasting cholesterol and triglycerides at baseline, 6 month and every 12 month thereafter Fasting glucose at baseline and 12 month	Serum creatinine at baseline if starting on TDF ALT at baseline and if symptomatic on NVP CD4 and VL at month 6, 1 year and every 12 month thereafter FBC or haemoglobin at baseline and month 1, 2, 3 and 6 if on AZT Creatinine at month 3 and 6 and every 12 month if on TDF Fasting cholesterol and triglycerides at 3 month if on LPV/r	Serum creatinine at baseline if starting on TDF ALT at baseline and if symptomatic on NVP CD4 at 1 year on ART VL at month 6, 1 year on ART and every 12 month FBC or haemoglobin at baseline and month 3 and 6 if on AZT Creatinine at month 3, 6, 1 year on ART and every 12 months if on TDF Fasting cholesterol and triglycerides at month 3 if on LPV/r	Serum creatinine at baseline if starting on TDF ALT at baseline and if symptomatic on NVP CD4 at 1 year on ART—once a CD4 reaches 200 cells/mm^3^ no further annual CD4 monitoring VL at month 6, 1 year on ART and every 12 month FBC or haemoglobin at baseline and month 3 and 6 if on AZT Creatinine at month 3, 6, 1 year on ART and every 12 months if on TDF Fasting cholesterol and triglycerides at start and month 3 if on LPV/r

South Africa uses HIV viral load testing to determine when a patient is considered to have failed an ART regimen. First-line treatment failure, defined as virologic failure, is defined as persistent viral loads ≥ 1000 copies/mL on two occasions within a 3 month interval [17–19]. Given low rates of primary NNRTI resistance in South Africa which are estimated to be less than 5% [20], lack of viral suppression after treatment initiation is generally related to poor adherence. In order to delay switching to more expensive regimens, patients with an elevated viral load (≥ 1000 copies/mL) receive intensified adherence counselling before repeating the viral load between 1 and 3 months later. If patients fail to suppress they are then switched to a PI-based second-line regimen with the drug lopinavir/ritonavir (LPV/r).

The management of patients failing second-line ART using intensified adherence counselling has been described before [21, 22]. Briefly, at Themba Lethu Clinic patients with elevated viral loads on second-line receive intensified adherence counselling followed by repeat viral load monitoring 2–4 months later. Patients who resuppress (< 400 copies/mL) continue to receive the standard of care. If they do not, patients are referred for ongoing adherence support. If the clinician decides adherence has improved and the viral load remains ≥ 1000 copies/mL or if resuppression is delayed (< 1 log reduction in viral load), resistance testing may be ordered [22]. Results of genotype tests, medical and social histories are reviewed and patients with PI resistance are recommended for third-line ART with an individualized regimen containing ETV, DRV/r and/or RAL. Third-line drugs are managed centrally by the NDoH through application to a national third-line committee and decisions to allow use are based on prior exposure, adherence and genotype results. Currently access to third-line drugs is limited to patients with PI-resistance.

At Themba Lethu Clinic, longitudinal clinical and demographic data are collected and stored on the electronic patient management system, TherapyEdge-HIV™. CD4 cell counts and viral load results are imported directly into the database from the National Health Laboratory Service (NHLS) each day. Death is ascertained through patient tracing and is often reported or confirmed by family. Where available (61%), patients national identification numbers are linked with the National Vital Registration System to confirm all-cause mortality/death [23, 24].

### Study population

As the study had two main objectives we analysed data from two overlapping study cohorts.

### Second-line cohort (predictors of switch to third-line)

To predict switch to third-line we included adult (≥ 18 years at the availability of third-line) patients who were on a PI-based second-line regimen as of 01 August 2012 at Themba Lethu Clinic. The analysis was restricted to patients with significant viraemia on second-line ART; those who had at least one viral load recorded at least 3 months after commencing second-line ART and had a viral load ≥ 1000 copies/mL on at least one occasion after the availability of third-line ART (01 August 2012) [25–27].

### Study variables

Current age, second-line regimen, viral load and CD4 count are defined as the value closest to 01 August 2012. Initiating second-line regimen was defined as the first PI-based regimen after April 2004 but before 01 August 2012. Time on second-line ART, calculated from start of second-line until 01 August 2012, was categorized at 96 weeks based on standardized reporting of outcomes at 0, 24, 48 and 96 weeks. History of Tuberculosis (TB) treatment was defined as a TB diagnosis recorded in the clinical conditions or the prescription of anti-TB medication (e.g. Rifafour^®^ or any of the standard first-line agents used in the treatment of TB; rifampicin, isoniazid, pyrazinamide, streptomycin, ethambutol etc.) after the start of second-line ART but before 01 August 2012. Current TB treatment was defined as a TB diagnosis recorded or the prescription of anti-TB medication after the start of second-line ART and is still being administered as of 01 August 2012.

### Statistical analysis

The primary outcome of interest among those with significant viraemia on second-line ART post 01 August 2012 was switch to third-line ART. For the second-line cohort, demographic and clinical characteristics are presented using proportions for categorical variables and medians with corresponding interquartile ranges (IQR) for continuous variables.

We considered the following viral load responses as potential predictors of switch to third-line ART: (i) never suppressed on second-line (defined as all viral load results ≥ 400 copies/mL between 3 months post second-line ART initiation and 01 August 2012); and (ii) viral load blips on second-line ART (0 vs. ≥ 1) (defined a viral load ≥ 1000 copies/mL preceded and followed by a viral load < 400 copies/mL; and restricted to patients with at least 3 viral loads between 3 months post second-line ART initiation and 01 August 2012). Additionally, we considered other potential predictors of switch to third-line ART such as regimen change, defined as any change in regimen from the patients’ initiating second-line regimen before 01 August 2012.

To assess predictors of switch to third-line ART we used Cox proportional hazards regression among those with significant viraemia on second-line ART post 01 August 2012. Follow-up time was calculated from 01 August 2012 (the availability of third-line ART), until the earliest of switch to third-line, death, loss to follow-up (LTF), transfer-out or close of dataset on 19 January 2017. Loss to follow-up was defined as being ≥ 3 months late for the last scheduled visit with no subsequent visit. Variables with a p value less than 0.1 in the univariate analysis and a priori variables (e.g. age and gender) were included in the final multivariate model. Because the number with the outcome of interest was limited (n = 36), the final model was restricted by the number of independent variables that could be added (i.e. one independent variable for every five to ten outcomes observed). All analyses were conducted using SAS version 9.3 (SAS Institute, Cary, North Carolina, USA).

### Third-line cohort

To describe characteristics of patients on third-line ART as well as their virologic outcomes, we included all adult patients (≥ 18 years at the availability of third-line; on third-line ART at Themba Lethu Clinic, defined as a regimen containing etravirine, ritonavir boosted darunavir or raltegravir between 01 August 2012 and 19 January 2016, allowing at least 12 months follow-up time for virologic response (i.e. close of dataset 19 January 2017). Medical file reviews were used to confirm the start date of third-line treatment. Clinical trial patients who transferred into Themba Lethu Clinic on a regimen containing one of the third-line drugs (e.g. ETV, DRV/r and RAL; n = 15) were excluded if available data confirmed that the clinical trial was not a third-line clinical trial (e.g. ACTG A5273, MK0518-292-00 and ACTG A5290).

### Study variables

For the third-line cohort CD4 count and viral load closest to the start of third-line ART were defined as a CD4 count and viral load result after 01 August 2012, but no more than 14 days post third-line initiation to allow for laboratory reporting and capturing when manual data entry was required. We categorized third-line patients into: (1) those that had initiated third-line ART at Themba Lethu (those who were switched to third-line ART via following national treatment guidelines); and (2) those that had been transferred into Themba Lethu Clinic on a third-line regimen (i.e. because they had initiated third-line elsewhere, possibly as a clinical trial patient, and transferred into the clinic on third-line). We also report the proportion of patients that started third-line in a clinical trial.

The primary outcome for the third-line cohort was viral load suppression on third-line ART; defined as a viral load < 400 copies/mL closest to 12 months post third-line initiation (± 3 months). We also estimate the proportion of patients virally suppressed (< 400 copies/mL) at the first follow-up viral load recorded, taken > 1 month but ≤ 6 months after the start of third-line ART (early suppression). Additionally, time to first ever suppressed viral load on third-line ART was defined as the time in months from 1 month post third-line ART initiation to first ever suppressed viral load (< 400 copies/mL) recorded within the 12 (± 3) months of follow-up from third-line initiation.

### Statistical analysis

For the third-line cohort, we again present demographic and clinical characteristics using proportions for categorical variables and medians with corresponding IQRs for continuous variables.

For patients who transferred-out to another facility after start of third-line ART, we reviewed the patient clinic file and recorded the transfer-out facility. Where possible, we traced patients (using their unique patient identification number on TherapyEdge-HIV™) to the transfer facility and recorded viral load results that were available. This allowed us to assign a viral load outcome to patients that were previously reported as transfer-out.

## Results

### Cohort descriptions

Among 719 patients in care, on second-line ART at Themba Lethu Clinic with significant viraemia after 01 August 2012, more than two-thirds were female (66.8%; 480/719) with a median age of 39.4 years (IQR 33.3–46.3). Most patients initiated on a second-line regimen of TDF, 3TC/FTC and LPVr (27.3%; 196/719), followed by zidovudine (AZT), AZT, 3TC/FTC and LPVr (22.7%; 163/719) and then AZT, LPVr and didanosine (ddI) (16.6%; 119/719) (Table 2). At start of follow-up (01 August 2012), the median (IQR) CD4 count was 415.0 (253.0–583.0) and the median (IQR) viral load was 249.5 (51.0–2791.5) copies/mL (Table 2).

**Table 2:** Characteristics of patients included in the second-line cohort (n = 719)

Characteristic at second-line initiation		Total (n = 719)	Switched to third-line (n = 36)	Patients remaining on second-line (n = 683)
Gender	N, %			
Male		239/719 (33.2%)	12/36 (33.3%)	227/683 (33.2%)
Female		480/719 (66.8%)	24/36 (66.7%)	456/683 (66.8%)
Current age, years	Median, IQR	39.4 (33.3-46.3)	40.0 (35.2-46.8)	39.3 (33.3–46.3)
< 30	N, %	92/719 (12.8%)	4/36 (11.1%)	88/683 (12.9%)
30–45		417/719 (58.0%)	20/36 (55.6%)	397/683 (58.1%)
≥ 45		210/719 (29.2%)	12/36 (33.3%)	198/683 (29.0%)
Current CD4 count, cells/mm^3^	Median, IQR	415.0 (253.0-583.0)	298.5 (183.0-397.0)	420.5 (255.0–592.0)
< 100	N, %	49/600 (8.2%)	4/26 (15.4%)	45/574 (7.8%)
100–200		61/600 (10.2%)	3/26 (11.5%)	58/574 (10.1%)
200–350		131/600 (21.8%)	8/26 (30.8%)	123/574 (21.4%)
≥ 350		359/600 (59.8%)	11/26 (42.3%)	348/574 (60.6%)
Missing		119	10	109
Current second-line regimen	N, %			
AZT + ddI + LPVr		7/719 (1.0%)	1/36 (2.8%)	6/683 (0.9%)
TDF + 3TC/FTC + LPVr		247/719 (34.4%)	10/36 (27.8%)	237/683 (34.7%)
AZT + 3TC + LPVr		259/719 (36.0%)	16/36 (44.4%)	243/683 (35.6%)
Other		206/719 (28.7%)	9/36 (25.0%)	197/683 (28.8%)
Current viral load, copies/mL	Median, IQR	249.5 (51.0-2791.5)	6510.0 (1505.0-32,718.0)	235.0 (49.0–1932.0)
< 1000	N, %	453/660 (68.6%)	8/33 (24.2%)	445/627 (71.0%)
1000–50,000		140/660 (21.2%)	19/33 (57.6%)	121/627 (19.3%)
≥ 50,000		67/660 (10.2%)	6/33 (18.2%)	61/627 (9.7%)
Missing		59	3	56
Year of second-line initiation	N, %			
2004–2010		313/719 (43.5%)	22/36 (61.1%)	291/683 (42.6%)
2010–2012		406/729 (56.5%)	14/36 (38.9%)	392/683 (57.4%)
Initiating second-line regimen	N, %			
AZT + ddI + LPVr		119/719 (16.6%)	10/36 (27.8%)	109/683 (16.0%)
TDF + 3TC/FTC + LPVr		196/719 (27.3%)	6/36 (16.7%)	190/683 (27.8%)
AZT + 3TC + LPVr		163/719 (22.7%)	6/36 (16.7%)	157/683 (23.0%)
Other		241/719 (33.5%)	14/36 (38.9%)	227/683 (33.2%)
Never suppressed on second-line ART prior to August 2012	N, %	90/595 (15.1%)	9/33 (27.3%)	81/562 (14.4%)
Missing		124	3	121
Viral load blips on second-line prior to August 2012	N, %			
0		317/488 (65.0%)	16/30 (53.3%)	301/458 (65.7%)
≥ 1		171/488 (35.0%)	14/30 (46.7%)	157/458 (34.3%)
Missing		231	6	225
Time to first viral load on second-line ART, days	Median, IQR	175.0 (125.0–252.0)	169.0 (118.0–280.0)	175.0 (126.0–244.0)
Total person time calculated from 01 August 2012, months	Median, IQR	53.5 (44.7–53.5)	12.4 (8.0–25.3)	53.5 (51.9–53.5)

*ABC* abacavir; *3TC* lamivudine; *FTC* emtricitabine; *LPVr* lopinavir ritonavir; *TDF* tenofovir; *AZT* zidovudine; *ddI* didanosine; *d4T* stavudine, *IQR* interquartile range

Among 719 patients with significant viraemia on second-line ART after 01 August 2012, 36/719 (5.0% over a median time of 54 months) switched to third-line ART. When looking at poor viral load response during second-line ART, a higher proportion of patients who switched to third-line (n = 36) experienced viral load blips on second-line ART (46.7% vs. 34.3%) or never suppressed on second-line ART (27.3% vs. 14.4%).

### Predictors of switch to third-line ART

While no differences were observed across gender, age groups and regimen change, we found that time on second-line prior to the availability of third-line ART, calculated from start of second-line until 01 August 2012, was a strong predictor of switch among those with significant viraemia (≥ 96 weeks vs. < 96; aHR 2.53 95% CI 1.03–6.22). When considering indicators of poor viral load response patients who never managed to suppress their viral load while on second-line therapy were more likely to switch to third-line ART (aHR 3.37 95% CI 1.47–7.73) (Table 3).

**Table 3:** Crude and adjusted hazard ratios of switch to third-line (n = 719)

Characteristics at second-line initiation	N, row %	Crude HR 95% CI	P value	aHR 95% CI	P value
Gender					
Female	24/480 (5.0%)	1.0		1.0	
Male	12/239 (5.0%)	1.01 (0.50–2.01)	0.99	1.09 (0.52–2.29)	0.81
Age, years					
18–30	8/156 (5.1%)	1.0		1.0	
30–45	20/407 (4.9%)	0.95 (0.42–2.15)	0.90	0.91 (0.39–2.11)	0.82
≥ 45	8/156 (5.1%)	0.95 (0.36–2.54)	0.93	0.89 (0.32–2.49)	0.82
Time on second-line (calculated from start of second-line until 01 August 2012), weeks					
< 96	9/317 (2.8%)	1.0		1.0	
≥ 96	27/402 (6.7%)	2.37 (1.11–5.03)	0.03*	2.53 (1.03–6.22)	0.04*
Regimen change prior to 01 August 2012					
No	20/493 (4.1%)	1.0			
Yes	16/226 (7.1%)	1.76 (0.91–3.39)	0.09		
Current CD4 count, cells/mm^3^					
< 100	4/49 (8.2%)	2.81 (0.90–8.83)	0.08		
100–200	3/61 (4.9%)	1.66 (0.46–5.96)	0.43		
200–350	8/131 (6.1%)	2.10 (0.85–5.23)	0.11		
≥ 350	11/359 (3.1%)	1.0			
Never suppressed on second-line ART prior to August 2012					
No	24/505 (4.8%)	1.0		1.0	
Yes	9/90 (10.0%)	2.32 (1.08–4.99)	0.03*	3.37 (1.47–7.73)	0.00*
Viral load blips on second-line ART prior to August 2012					
0	16/317 (5.1%)	1.0			
≥ 1	14/171 (8.2%)	1.64 (0.80–3.36)	0.18		
History of TB					
No	30/658 (4.6%)	1.0		1.0	
Yes	6/61 (9.8%)	2.29 (0.95–5.50)	0.06	1.99 (0.81–4.85)	0.13
Currently on TB treatment					
No	34/694 (4.9%)	1.0			
Yes	2/25 (8.0%)	1.75 (0.42–7.27)	0.44		

*ABC* abacavir, *3TC* lamivudine, *FTC* emtricitabine, *LPVr* lopinavir/ritonavir, *TDF* tenofovir, *AZT* zidovudine, *ddI* didanosine, *d4T* stavudine, *HR* hazard ratio, *aHR* adjusted hazard ratio; * p < 0.05

### Characteristics of patients on third-line ART at Themba Lethu Clinic

Table 4 presents the characteristics and early outcomes of patients on third-line ART. From 01 August 2012 to 19 January 2016, 82 patients received third-line ART treatment at Themba Lethu Clinic. Just over half of them (63.4%, 52/82) initiated a third-line regimen containing ritonavir boosted darunavir—alone or in combination with raltegravir or etravirine. Close to half (42.7%; 35/82) had a regimen containing raltegravir, alone or in combination with other drugs. Half of patients (50.0%) had a regimen containing four or more drugs and 24.4% had a regimen containing five or more drugs. 43.9% (36/82) had a TDF or TDF and AZT NRTI backbone.

**Table 4:** Third-line regimen patient characteristics and early outcomes on third-line ART

Characteristic at start of third-line		Total (n = 82)	Switched to third-line (n = 36)	Transferred in on third-line ART (n = 46)
Gender	N, %			
Male		31/82 (37.8%)	12/36 (33.3%)	19/46 (41.3%)
Female		51/82 (62.2%)	24/36 (66.7%)	27/46 (58.7%)
Age, years	Median, IQR	40.0 (35.5–47.3)	40.0 (35.2–46.8)	40.0 (36.2–47.3)
18–30	N, %	5/82 (6.1%)	4/36 (11.1%)	1/82 (2.2%)
30–45		52/82 (63.4%)	20/36 (55.6%)	32/82 (69.6%)
≥ 45		25/82 (30.5%)	12/36 (33.3%)	13/82 (28.3%)
CD4 count closest to start of third-line ART, cells/mm^3^	Median, IQR N, %	277.0 (194.0–439.0)	263.0 (156.0–390.0)	339.0 (212.0–472.0)
< 100		9/58 (15.5%)	5/27 (18.5%)	4/31 (12.9%)
100–200		6/58 (13.7%)	3/27 (11.1%)	3/31 (9.7%)
200–350		21/58 (32.9%)	12/27 (44.4%)	9/31 (29.0%)
≥ 350		22/58 (35.6%)	7/27 (25.9%)	15/31 (48.4%)
Missing		24/82	9/36	15/46
Third-line regimen	N, %			
DRV/r + RAL + ETV		0/82 (0.0%)	0/36 (0.0%)	0/46 (0.0%)
DRV/r + RAL		17/82 (20.7%)	11/36 (30.6%)	6/46 (13.0%)
RAL + ETV		0/82 (0.0%)	0/36 (0.0%)	0/46 (0.0%)
DRV/r + ETV		5/82 (6.1%)	2/36 (5.6%)	3/46 (6.5%)
DRV/r		30/82 (36.6%)	12/36 (33.3%)	18/46 (39.1%)
RAL		18/82 (22.0%)	4/36 (11.1%)	14/46 (30.4%)
ETV		12/82 (14.6%)	7/36 (19.4%)	5/46 (10.9%)
NRTI backbone				
TDF		31/82 (37.8%)	13/36 (36.1%)	18/46 (39.1%)
AZT		4/82 (4.9%)	1/36 (2.8%)	3/46 (6.5%)
ABC		0/82 (0.0%)	0/36 (0.0%)	0/46 (0.0%)
TDF + AZT		5/82 (6.1%)	4/36 (11.1%)	1/46 (2.2%)
TDF + ABC		0/82 (0.0%)	0/36 (0.0%)	0/46 (0.0%)
Other		42/82 (51.2%)	18/36 (50.0%)	24/46 (52.2%)
Viral load closest to start of third-line ART, copies/mL (> 400 copies/mL)	Median, IQR N, %	13,930.0 (2655.0–62,515.0)	24,096.5 (3877.5–84,934.0)	7883.0 (1504.0–41,485.0)
< 1000		11/73 (15.1%)	3/36 (8.3%)	8/37 (21.6%)
1000–50,000		40/73 (54.8%)	20/36 (55.6%)	20/37 (54.1%)
≥ 50,000		22/73 (30.1%)	13/36 (36.1%)	9/37 (24.3%)
Missing		9/82	0/36	9/46
Year starting third-line ART	N, %			
2012		20/82 (24.4%)	3/36 (8.3%)	17/46 (37.0%)
2013–2016		62/82 (75.6%)	33/36 (91.7%)	29/46 (63.0%)
Time to first ever viral suppression on third-line	N, %			
1–6 months		49/82 (59.8%)	22/36 (61.1%)	27/46 (58.7%)
6–12 months		16/82 (19.5%)	7/36 (19.4%)	9/46 (19.6%)
Missing		17/82 (20.7%)	7/36 (19.4%)	10/61 (21.7%)
Outcomes on third-line ART				
Viral suppression on third-line by first viral load within 6 months (early suppression)	N, %			
Alive, in care and suppressed		47/82 (57.3%)	20/36 (55.6%)	27/46 (58.7%)
Alive, in care and not suppressed		13/82 (15.9%)	7/36 (19.4%)	6/46 (13.0%)
Alive, in care and no repeat viral load/no repeat viral load within follow-up		17/82 (20.7%)	7/36 (19.4%)	10/46 (21.7%)
Not alive or in care				
Died		2/82 (2.4%)	1/36 (2.8%)	1/46 (2.2%)
Loss to follow-up*		0/82 (0.0%)	0/36 (0.0%)	0/46 (0.0%)
Transfer-out		3/82 (3.7%)	1/36 (2.8%)	2/46 (4.4%)
Viral suppression on third-line by 12 months	N, %			
Alive, in care and suppressed		35/82 (42.7%)	16/36 (44.4%)	19/46 (41.3%)
Alive, in care and not suppressed		7/82 (8.5%)	5/36 (13.9%)	2/46 (4.4%)
Alive, in care and no repeat viral load/no repeat viral load within follow-up		30/82 (36.6%)	12/36 (33.3%)	18/46 (39.1%)
Not alive or in care				
Died		4/82 (4.9%)	2/36 (5.6%)	2/46 (4.4%)
Loss to follow-up*		1/82 (1.2%)	0/36 (0.0%)	1/46 (2.2%)
Transfer-out		5/82 (6.1%)	1/36 (2.8%)	4/46 (8.7%)
Never suppressed viral load below 400 copies/mL by 12 months	N, %	6/71 (8.5%)	4/33 (12.1%)	2/38 (5.3%)

*ABC* abacavir; *3TC* lamivudine; *LPVr* lopinavir ritonavir; *TDF* tenofovir; *AZT* zidovudine; *ddI* didanosine; *d4T* stavudine, *ETR* etravirine, *RAL* raltegravir, *DRV/r* ritonavir boosted darunavir, *IQR* interquartile range

* Loss to follow-up defined as missing their last scheduled visit ≥ 3 months

Of those on third-line, 37.8% were male (n = 31) and the median age at third-line initiation was 40.0 years (IQR 35.5–47.3). At third-line ART initiation, the CD4 count was 277.0 cells/mm^3^ (IQR 194.0–439.0) and median viral load was 13,930.0 copies/mL (IQR 2655.0–62,515.0).

Patients on third-line ART who were referred into Themba Lethu Clinic (n = 46) were similar to those that initiated second-line ART and switched to third-line ART at Themba Lethu Clinic in terms of the characteristics described in Table 4, aside from a high proportion of male patients (41.3 vs. 33.3%), lower proportion of patients aged 18–30 years (2.2 vs. 11.1%), higher proportion of patients with CD4 cell counts ≥ 350 cells/mm^3^ (48.4 vs. 25.9%), lower median viral load (7883.0 vs. 24,096.5 copies/mL) around the first recorded date of third-line ART as well as year starting third-line ART (2012; 37.0 vs. 8.3%, 2013–2016; 63.0 vs. 91.7%). Of the 46 patients identified to be referrals to third-line therapy (i.e. transferred into Themba Lethu Clinic on third-line), 10 (21.7%) had initiated treatment in a clinical trial (Additional file 1: Table S1).

At the end of follow-up the majority of patients were alive, in care (87.8%, n = 72/82) while 6.1% had transferred out (n = 5/82), 1.2% were loss to follow-up (n = 1/82) and 4.9% had died (n = 4/82) (Table 4). Of those that transferred out, tracing initiatives yielded viral load results for 4 of the 5 patients, with 4 patients (80%; 4/5) achieving viral suppression within 12 months of starting third-line. Of all patients in care and on third-line ART, 61.0% (47/77) and 48.6% (35/72) suppressed their viral load at 6 and 12 months, respectively. By tracing patients that transferred out (n = 5), this increased to 50.7% (39/77) by the end of follow-up. However, among all patients in care and on third-line ART with a viral load measured at 6 and 12 months, 78.3% (47/60) and 83.3% (35/42) suppressed their viral load respectively.

Among the 36 patients that initiated third-line ART at Themba Lethu, 44.4% (95% CI 29.0–60.8%) were virally suppressed (n = 16/36) by 12 months after third-line ART initiation. This was higher than the 41.3% (95% CI 27.8–55.9%) suppression among referral patients (n = 19/46).

## Discussion

As ART programmes in sub-Saharan Africa mature, increasing numbers of people will require third-line regimens. Here we report that 5% of patients with significant viraemia on second-line ART at Themba Lethu Clinic switched to third-line ART (over a median time of 54 months). The small proportion in need of third-line is consistent with what has been previously reported [6].

Implementation of third-line ART is resource intensive and requires more expensive drugs, access to resistance testing and an individualized approach to therapy where drug history, treatment toxicities and resistance patterns of patients are all taken into account. In a recent study of 400 patients with significant viraemia on second-line (viral load ≥ 1000 copies/mL) at Themba Lethu, the majority (65%) were able to re-suppress their viral load within 6 months following an adherence intervention while only 6% required third-line ART [22, 28]. Studies have shown the benefits of intensive adherence counselling among patients failing second-line ART, with patients re-suppressing following intensive counselling and adherence support, subsequently avoiding switching to more expensive third-line regimens [29].

A high HIV RNA at baseline or start of second-line ART has been demonstrated by others as a strong independent predictor of second-line failure [30–33]. In addition to a high burden of virus at the start of second-line ART, here we report that patients who never reach virologic suppression while on second-line are at increased risk of switch to third-line (aHR 3.37 95% CI 1.47–7.73). We also report that patients on second-line ART ≥ 96 weeks to be two and half time more likely to switch to third-line therapy (aHR 2.53 95% CI 1.03–6.22). Contrary to other reports we did not find that patients on TB treatment or those with a history of TB treatment were at increased risk of treatment failure or switch [25]. Reports have shown that Rifampicin, the cornerstone of anti-TB therapy, can reduce the PI tough concentration by more than 90% [34, 35]. Even though TB treatment was not identified as a significant predictor in this study, it should be noted that TB treatment may be a significant clinical contributing factor, especially if the dose of ritonavir is not adjusted to account for the reduced PI concentration associated with concomitant rifampicin [36].

Studies have demonstrated that patients who have suboptimal adherence to first-line ART are more likely to have suboptimal adherence to second-line ART and probably also third-line ART [37]. Therefore, these individuals should be targeted for interventions to improve adherence during first-line ART to avoid failure and switching to more expensive regimens in the future. Sub-optimal adherence and drug resistance may all be contributing to the increased risk of treatment failure and the need for third-line ART. A study conducted by Cox and colleagues among second-line ART patients with an elevated viral load of more than 400 copies/mL in a large informal settlement outside Cape Town in South Africa, suggests that patients who re-suppress at the first follow-up viral load test are three times more likely to remain virally suppressed, compared to those who reach viral re-suppression later (aHR 3.15 95% CI 2.2–4.4). The same may be true for viral suppression after starting second-line ART, highlighting that the main focus for patients failing second-line should be on adherence support [38]. Our study did not include genotypic resistance data, but supports findings of other studies which have reported that poor adherence, rather than drug resistance, as a major determinant of virologic failure among patients on second-line ART [30].

Compared to those who remained on second-line and did not switch, we show that patients who switched to third-line had lower CD4 counts at the start of follow-up (01 August 2012) (< 100 cells/mm^3^; 15.4% vs. 7.8%). Others have also shown that lower CD4 count at start of second-line predicts failure to achieve viral suppression on second-line ART [39, 40]. While no discernible differences were observed across gender, some reports suggest females are generally at higher risk of failure and switch to third-line [6, 41], while findings from resource-limited settings report male gender to be associated with these outcomes [42–44]. In our setting however, the risk for significant viraemia and switch to third-line was similar for males and females.

Of all patients receiving third-line ART at Themba Lethu Clinic, we found 42.7% (35/82; 95% CI 32.3–53.6%) suppressed their viral load on third-line ART by 12 months (35/72; 48.6% 95% CI 37.2–60.1 among those alive and in care at 12 months and 35/42; 83.3% 95% CI 69.8–92.4 among those alive and in care at 12 months with a viral load measured at this time). More specifically, majority of third-line patients (57.3% of all patients or 47/82; 61.0% among those alive and in care at 6 months and 47/60; 78.3% among those alive and in care at 6 months with a viral load measured at this time) achieved viral suppression in the first 6 months (early suppression). Time to first ever suppressed viral load while on third-line was relatively short with two-thirds of patients achieving their first ever suppressed viral load within 6 months of third-line initiation (59.8%). These results are consistent with those reported from Mumbai, India where most (61%; 11/18) patients achieved virologic suppression after a median duration of 6 months (183 days) on third-line ART [26]. Virologic suppression < 400 copies/mL has been reported as high as 83% among patients on third-line ART in South Africa (n = 152), however the study was conducted among patients on salvage ART in a Southern African private sector disease management program [5].

Moderate to high rates of loss to follow up (1.2%) and transfer-out (6.1%) respectively, may have restricted repeat viral load testing and subsequently underestimated suppression rates. To overcome this we managed to trace and obtain viral load outcomes for four of the five transfer-out patients. All four patients with viral load results were suppressed. For those patients with viral load data between 1 and 12 months of starting third-line (n = 71), 8.5% (95% CI 3.5–16.7; 6/71) never suppressed their viral load below 400 copies/mL.

This study has several limitations in addition to those already mentioned. We included patients on second-line ART as of 01 August 2012, when third-line became available, which may have introduced selection bias. Some potential predictors of treatment failure such as measures of adherence and routine resistance testing were not assessed in this study. However, the examination of more proximal determinants of significant viraemia (duration of current therapy and virologic response) may be important to evaluate, particularly in resource-limited settings where robust measures may not be readily available. Additionally, after March 2012 the standard of care for patients with an elevated viral load while on second-line therapy has improved with more intensified adherence counselling, follow-up and more frequent visits with experienced medical officers [28]. Our results may therefore not be generalizable to all public sector HIV clinics which rely on standard adherence counselling offered by a social worker/counsellor or may not have any resources devoted to adherence counselling. Although alive and in care post third-line ART initiation, 30/82 (36.6%) patients had no viral loads recorded, thus the proportion reported to be suppressed may be an underestimate due to missing data. We report that 22.0% of patients who received third-line contained a single third-line drug of RAL alone. This does not seem appropriate for a third-line regimen in patients with resistance to other agents and suggests that data may be incomplete. The limitations of routine data are well-described [45]. For transfer-in and transfer-out patients we could only trace these patients to the neighbouring HIV clinical trials unit, which also uses TherapyEdge-HIV™, where electronic medical records were linked using the unique patient identification number or TE number. Additionally, for patients who transferred in on a third-line regimen, treatment information preceding the date of transfer such as participation in a clinical trial patient or time on third-line are not available.

## Conclusion

Our results show that the need for third-line, based on significant viraemia while on second-line ART, is low (5% over a median time of 54 months). But that patients’ who switch to third-line ART have good early treatment outcomes and are able to suppress their viral load. Adherence counselling and resistance testing should be prioritized for patients that are at risk of failure, in particular those who never suppress on second-line and those who have been on PI-based regimen for extended periods. Despite a poor response to second-line ART, we report 57.3% (47/82) of all patients on third-line ART suppressed their viral load during the first 6 months on third-line therapy. In addition, tracing initiatives confirmed that 93.9% (77/82) of patients on third-line remained alive and in care by 12 months.

## Additional file

**Additional file 1: Table S1.** Summary of third-line patients transferred in from clinical trials (n=10).

## Abbreviations

RLS: resource-limited setting
ART: antiretroviral therapy
NNRTI: non-nucleoside reverse transcriptase inhibitor
PI: protease inhibitor
WHO: World Health Organization
ETV: etravirine
DRV/r: ritonavir boosted darunavir
RAL: raltegravir
NDoH: National Department of Health
LPV/r: lopinavir/ritonavir
NHLS: National Health Laboratory Services
TB: tuberculosis
IQR: interquartile range
LTF: loss to follow-up
AZT: zidovudine
ddI: didanosine
