# Supplementary material for: Predictors of switch to and early outcomes on third-line antiretroviral therapy at a large public-sector clinic in Johannesburg, South Africa
Source: AIDS Res Ther. 2018 Apr 10;15:10. doi: 10.1186/s12981-018-0196-9 (PMC5891887; doi:10.1186/s12981-018-0196-9)
Supplement: Supplementary file 1 — Additional file 1: Table S1. Summary of third-line patients transferred in from clinical trials (n=10). [file 12981_2018_196_MOESM1_ESM.docx]

**Additional file 1: Table S1. Summary of third-line patients transferred in from clinical trials (n=10)**

| **Clinical Trial** | **Description** | **N, %** |
| --- | --- | --- |
| ACTG A5288: Third-line ART in Resource Limited Settings | Protocol A5288 was launched in 2013 to compare three strategies for study participants who have failed first and second-line antiretroviral treatment in resource limited settings. | 5/10 (50.0%) |
| START trial: The Strategic Timing of Antiretroviral Treatment | This trial was a large scale international multi-country, multi-site randomized clinical trial which examined the timing of ART initiation based on differing CD4 cell count thresholds. | 2/10 (20.0%) |
| TMC125IFD3002 | An open-label study to evaluate the safety, tolerability and pharmacokinetics of ETR in combination with other ARVs in ARV treatment-experienced HIV infected patients. Primary outcomes of this trial was the proportion of patients with adverse events (AEs), while secondary outcomes included the proportion of patients virologically suppressed as well as change in CD4 cell count. | 2/10 (20.0%) |
| TMC125-TiDP35-C239: Continued Access to Etravirine (ETR) in Treatment Experienced HIV-1 Infected Children and Adolescents | The aim of this trial was to follow up patients for safety measurements until ETR was commercially available. | 1/10 (10.0%) |
